# Extraneural Soft Tissue Perineurioma: A Report of a Rare Case of Peripheral Nerve Sheath Tumor

**DOI:** 10.7759/cureus.73169

**Published:** 2024-11-06

**Authors:** Ramki Arunachalam Ganesh, Karthikeyan Selvaraj, Srinivasan Chandran, Jesu Pencilin Yesuvadiyan

**Affiliations:** 1 General Surgery, Sree Balaji Medical College and Hospital, Chennai, IND

**Keywords:** benign peripheral nerve sheath tumor, benign tumor, extraneural perineurioma, perineurioma, soft tissue tumor

## Abstract

Extraneural perineuriomas are rare, benign soft tissue tumors arising from perineurial cells, which form the protective lining of peripheral nerves. These tumors are infrequently encountered in the foot, posing diagnostic challenges due to their rarity and non-specific clinical presentation. Here, we describe the case of a 45-year-old woman, who had a swelling over the right foot dorsum for four years for which an excision biopsy was done. Her histopathological variant and immunohistochemistry were consistent with extraneural perineurioma. This article aims to present a comprehensive review of extraneural perineuriomas, focusing on a case study involving the foot, and to discuss the clinical and histopathological characteristics, differential diagnosis, treatment options, and prognosis of this uncommon entity.

## Introduction

Peripheral nerve sheath tumors (PNSTs) encompass a number of benign and malignant tumors, with neurofibromas and schwannomas being the most frequently encountered types. Extraneural perineurioma, a rare subtype of PNST, arises from perineurial cells found outside the nerve itself [1]. Despite being rare, it differs from other soft tissue tumors due to its distinct clinical and histopathological characteristics. Extraneural perineurioma is a rare entity, with most cases reported sporadically. According to the literature, it comprises less than 1% of all soft tissue tumors. Cases have been reported in people of all ages, from children to the elderly, with no evident demographic preference. In this article, we describe the case of a 45-year-old woman, who had a swelling over the right foot dorsum for four years for which an excision biopsy was done. The histopathological examination was consistent with features of extraneural perineurioma.

## Case presentation

**Figure 1:**
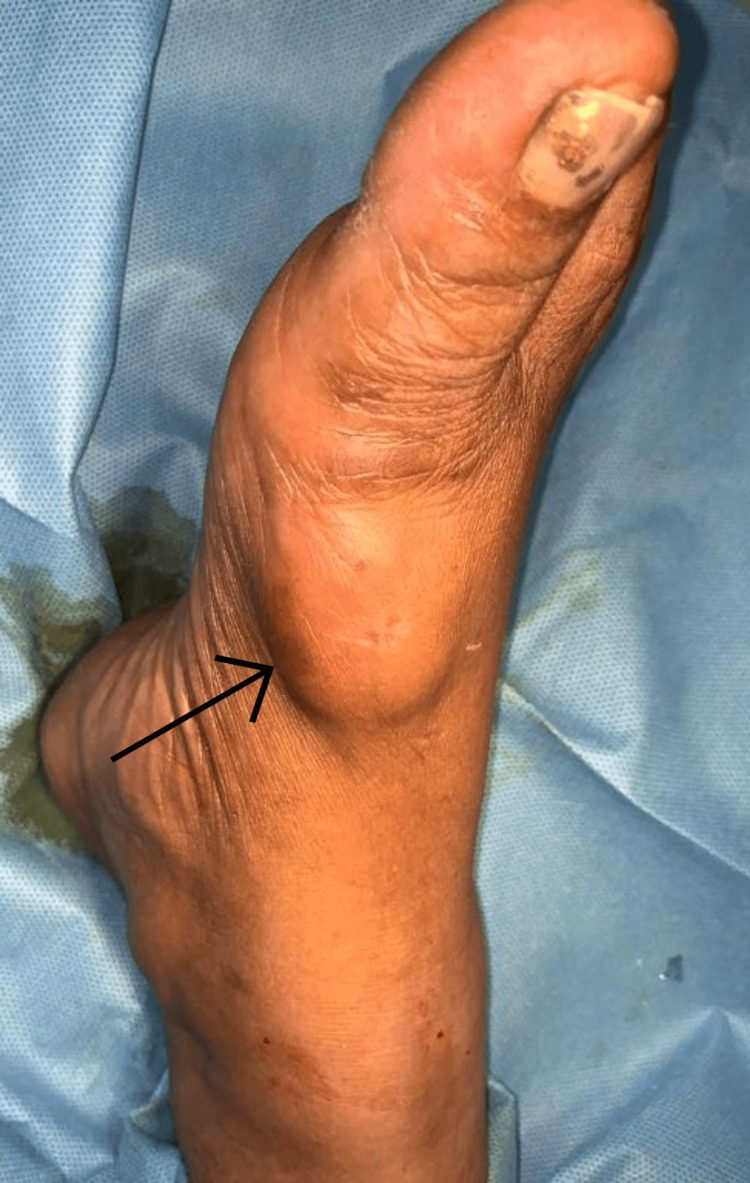
Clinical picture of the swelling over the first metatarsal region of the patient's right foot (marked with a black arrow)

A 45-year-old woman complained of painless swelling over the first metatarsal area of her right foot dorsum for more than four years when she presented to the surgical outpatient department. The onset of the swelling was subtle, initially small in size and increased over time to attain its current size. There was no prior history of trauma. There was no other similar swelling anywhere else in the body. There was no prior history of similar swelling. There was no history of previous surgeries. The patient has no comorbidities. On examination, a swelling of size approximately 4×3 cm (Figure 1) was noted over the dorsum of the right foot over the first metatarsal region.

**Figure 2:**
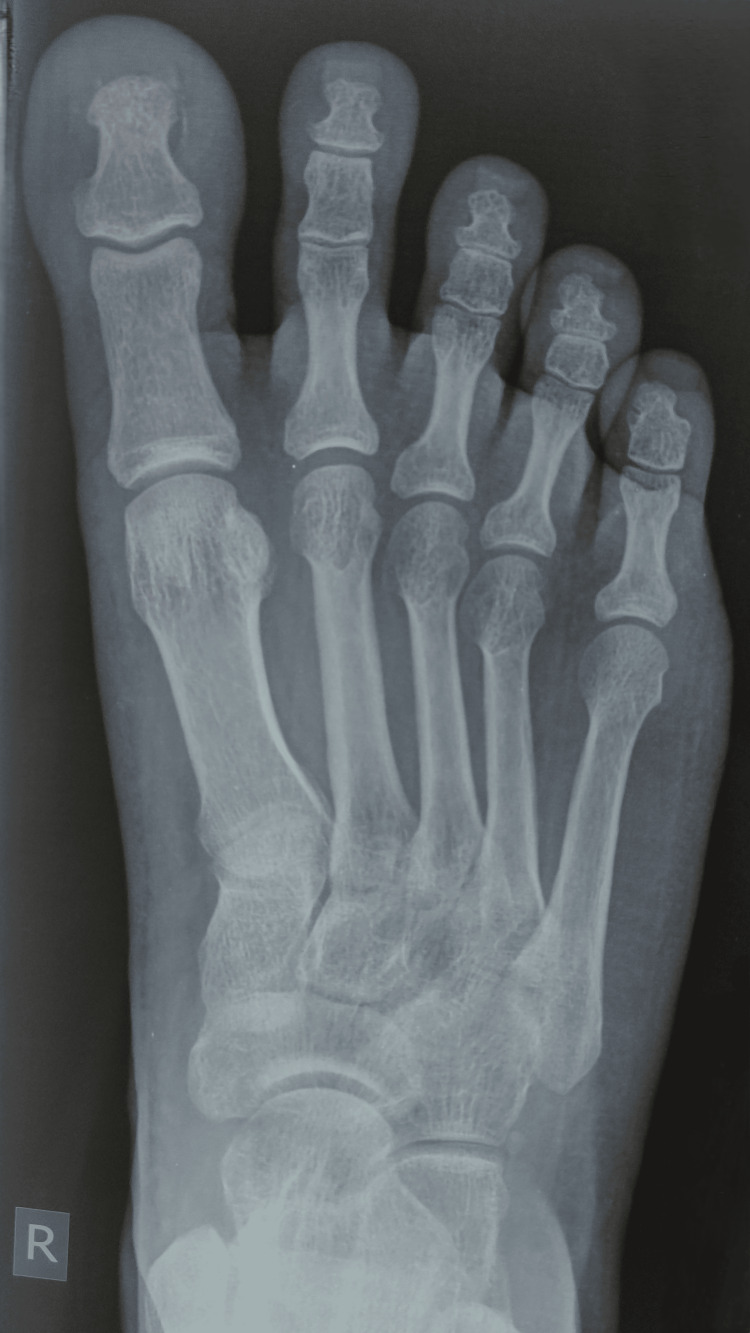
X-ray of the patient's right foot exhibiting no bony abnormalities

The swelling had limited side-to-side mobility with respect to the extensor hallucis longus (EHL) tendon and non-tender, and the skin over the swelling was pinchable with no discoloration or discharge. There was no edema or induration surrounding the swelling. Musculoskeletal, vascular, and neurological examinations were unremarkable. Her laboratory investigations such as complete blood count, erythrocyte sedimentation rate, bleeding time, and clotting time sent were within normal limits. Her ultrasound of the swelling showed a well-defined, smooth-marginated, hypoechoic lesion with no evidence of bony erosion or calcification. Her X-ray of the foot showed no fractures, no calcification, or any other periosteal reaction or osteomyelitis (Figure 2).

**Figure 3:**
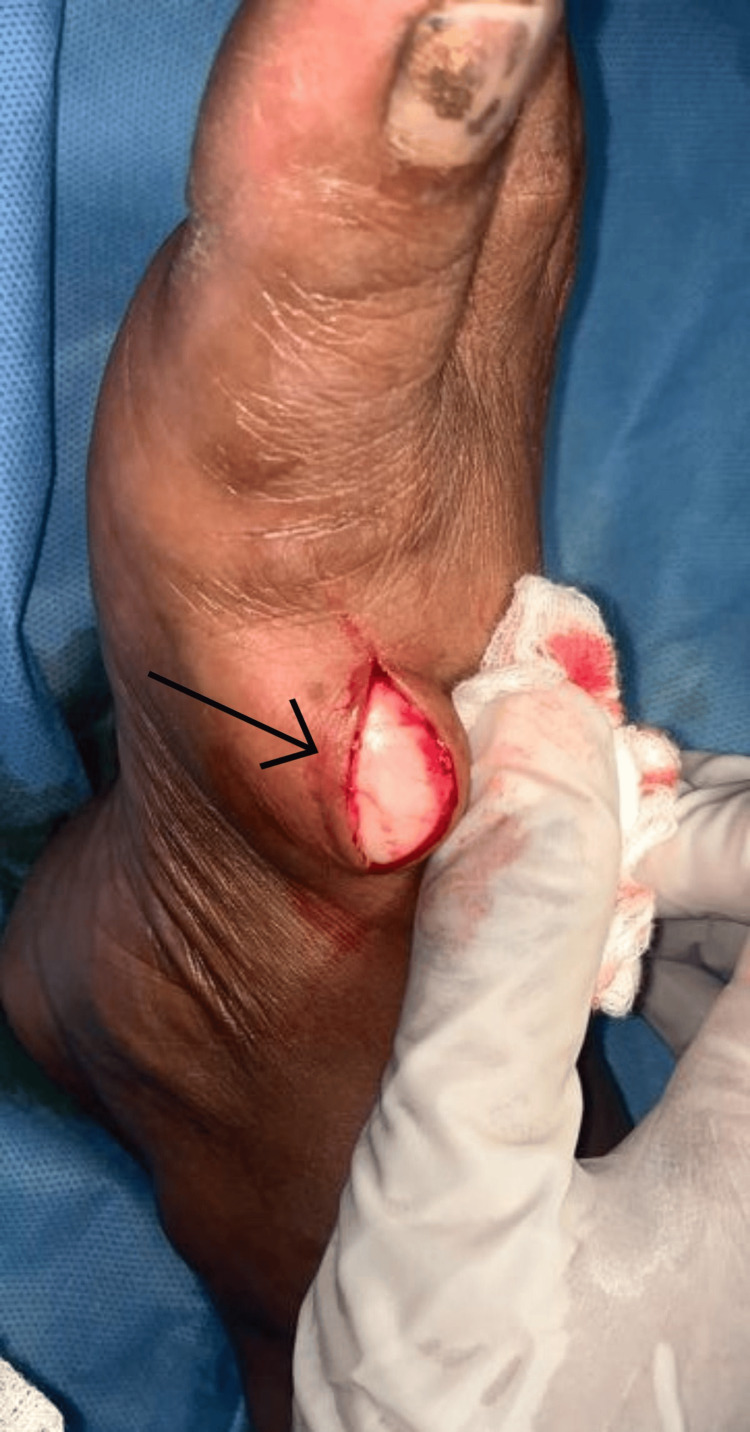
Intraoperative image showing the soft tissue lesion (marked with a black arrow)

**Figure 4:**
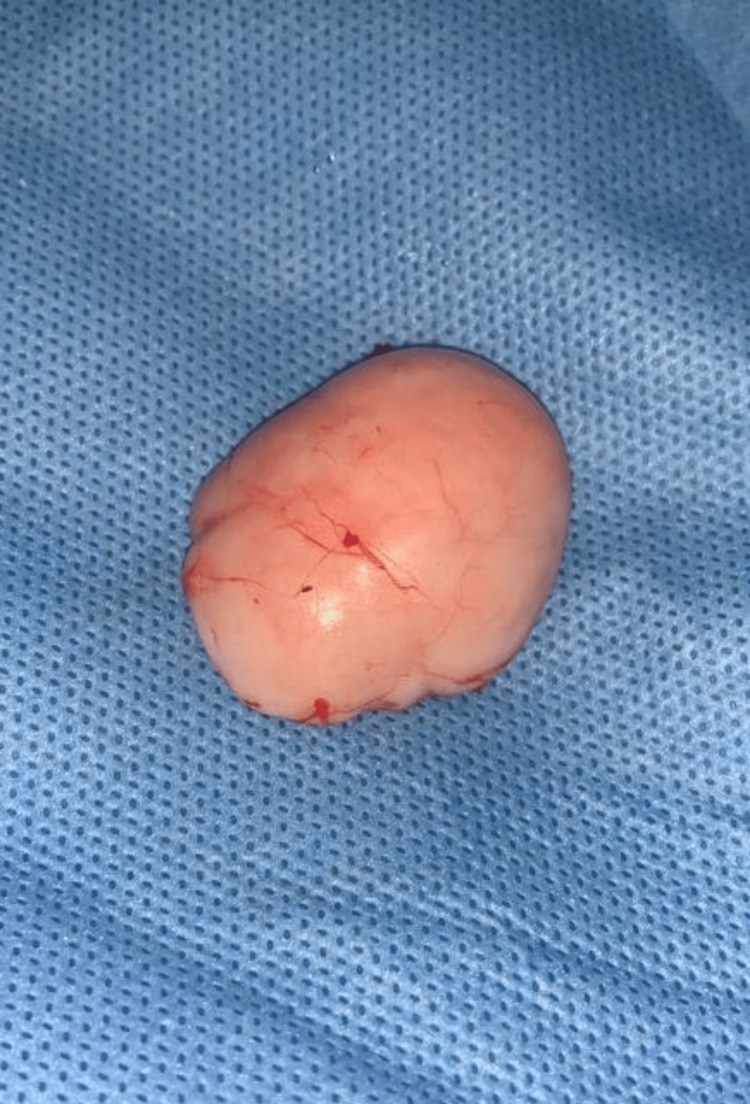
Image showing the excised soft tissue lesion

Fine-needle aspiration cytology (FNAC) of the lesion showed spindle cells. Excision biopsy was advised for the patient. The patient was taken up for an excision biopsy under local anesthesia after obtaining informed and written consent. A linear incision was placed over the skin above the swelling. The incision was deepened by blunt dissection. A firm, greyish-white mass originating near the EHL tendon was revealed (Figure 3). The mass excised in toto was approximately 4×3×1.5 cm in size (Figure 4) and was sent for histopathological examination. The EHL tendon was found to be intact. The wound was thoroughly washed with normal saline. After ensuring complete hemostasis, the skin was sutured with 3-0 Ethilon.

**Figure 5:**
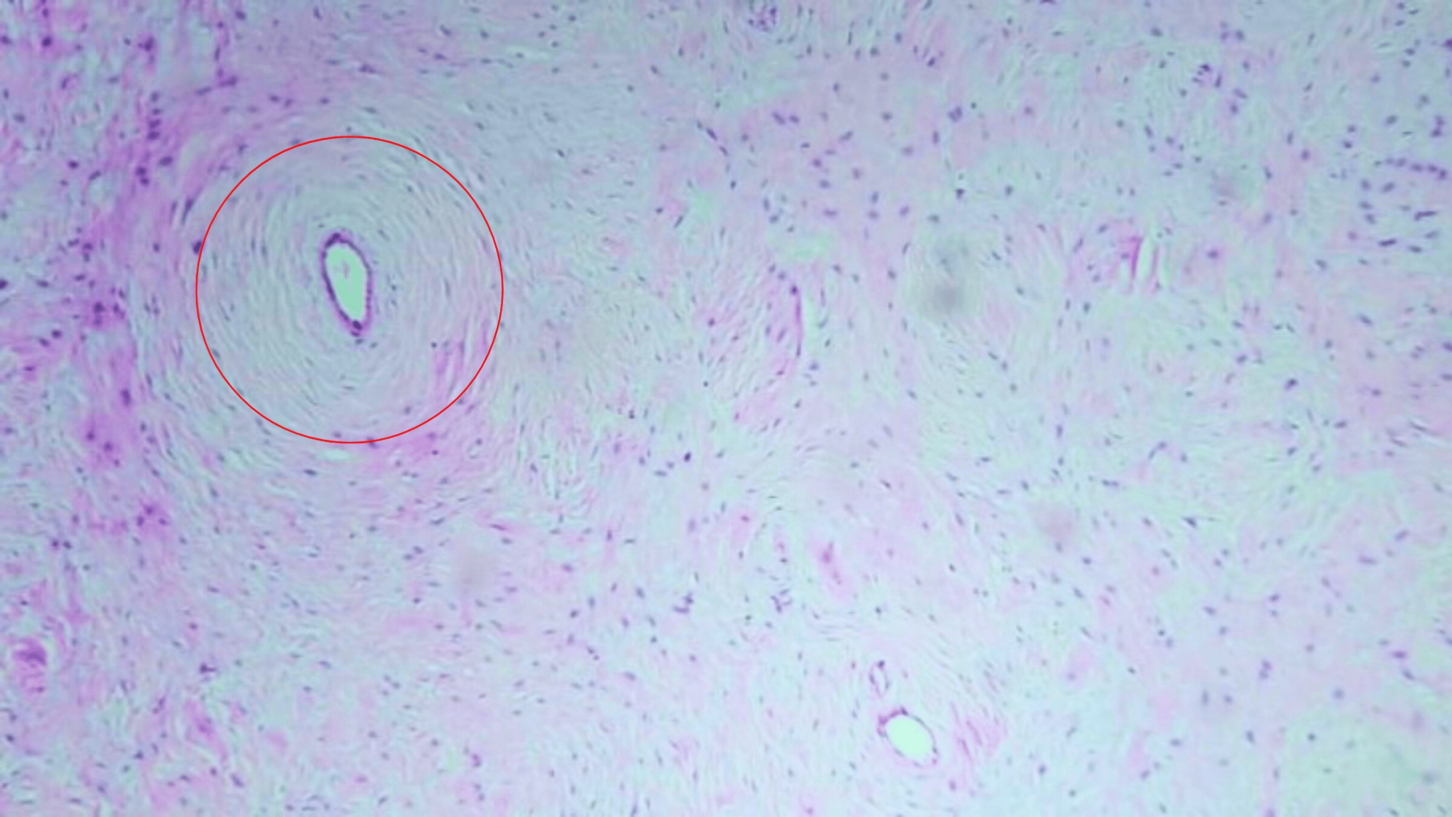
Low-power histopathological slide with hematoxylin and eosin stain showing slender spindle cells in a short whorl-like pattern (marked with a red circle)

**Figure 6:**
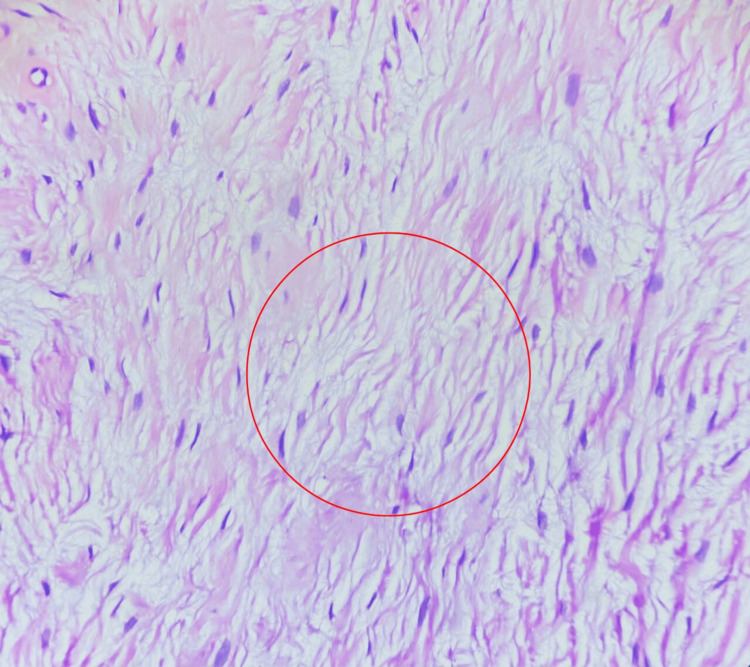
High-power histopathological slide with hematoxylin and eosin stain showing slender spindle cells (marked with a red circle)

The histopathological examination of the lesion showed a well-defined proliferation of slender spindle cells in a short whorl-like pattern, consistent with soft tissue extraneural perineurioma (Figure 5 and Figure 6).

**Figure 7:**
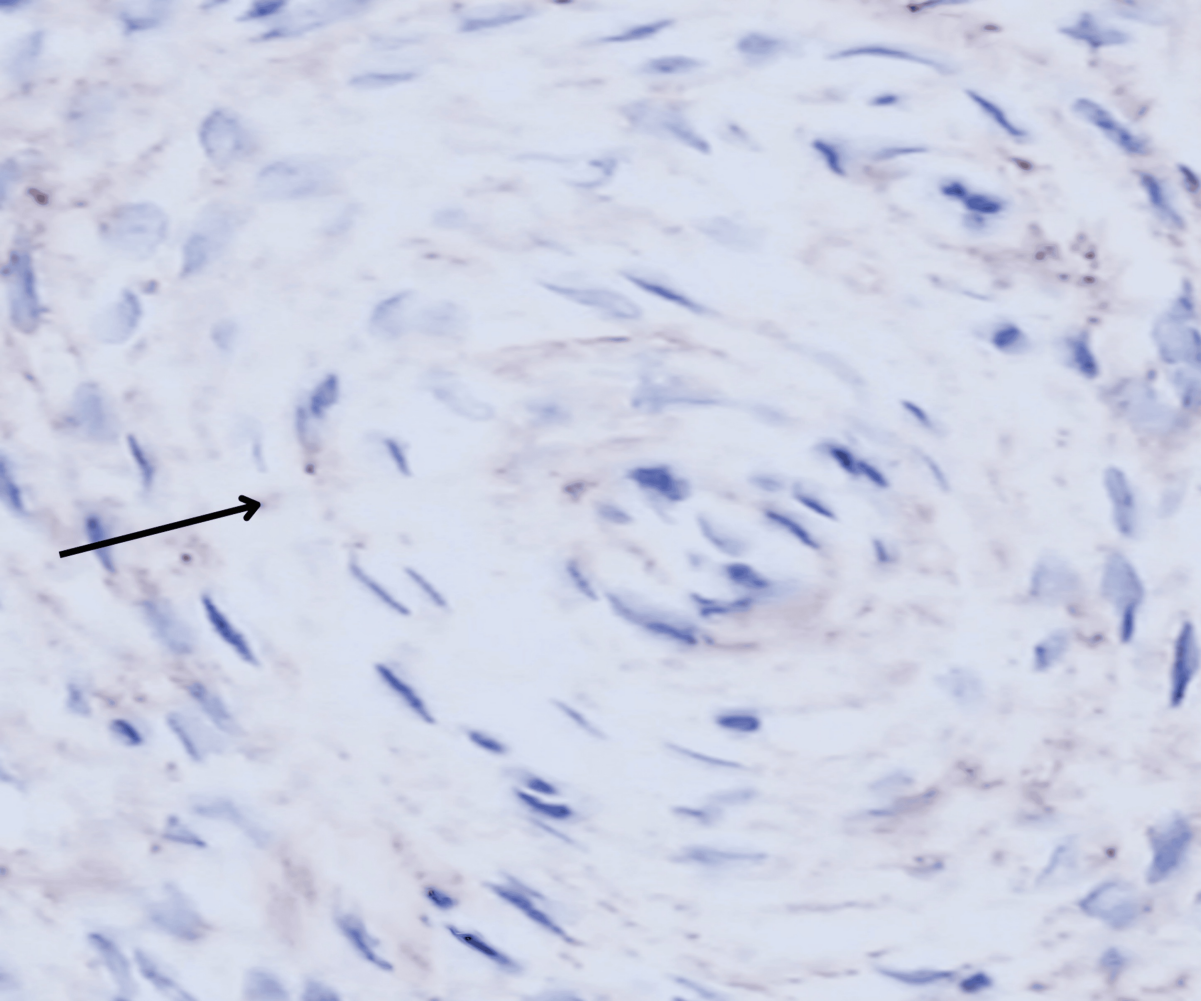
High-power immunohistochemistry staining for epithelial membrane antigen showing positivity (marked with a black arrow)

The lesion's immunohistochemistry (IHC) revealed that it was positive for epithelial membrane antigen (EMA) (Figure 7) but negative for S-100, CD34, desmin, and smooth muscle actin, ruling out schwannoma, dermatofibrosarcoma protuberans, and myoepithelial tumors, and was typical of extraneural perineurioma. The results of IHC markers are given in Table 1.

**Table 1:** Immunohistochemistry results

Immunohistochemical marker	Result
Epithelial membrane antigen	Positive
Desmin	Negative
Smooth muscle actin	Negative
Claudin-1	Negative
Protein S-100	Negative
CD34	Negative

The postoperative course of the patient was uneventful, and there was no sensory or motor neurological deficit or any other complications. There was no sign of tumor recurrence 12 months after the procedure.

## Discussion

Perineuriomas are uncommon benign tumors that primarily affect the peripheral nerves, with an affinity towards the extremities. In 1978, Lazarus and Trombetta gave the first description of it. Perineuriomas are traditionally categorized into two distinct types. Intraneural perineurioma is a type of tumor that originates directly within the nerve trunk. Extraneural perineurioma develops as a well-defined subcutaneous or, in rare cases, deep soft tissue or cutaneous mass that is not associated with a discernible nerve [2-4]. Extraneural perineuriomas are more prevalent than intraneural perineuriomas. The exact etiology of extraneural perineuriomas remains unclear, but genetic factors and prior trauma have been suggested as potential contributing factors [5,6].

Clinically, extraneural perineuriomas typically present as painless, slow-growing masses. They can occur at any anatomical site but are most commonly found in the extremities. The foot is an unusual location, making diagnosis challenging [7]. The differential diagnosis for soft tissue masses in the foot includes benign conditions such as fibromas, lipomas, and schwannomas, as well as malignant tumors like malignant MPNSTs and synovial sarcoma [8].

The cellular composition of neurofibromas and schwannomas, two of the most prevalent tumors of the peripheral nervous system, allows for differentiation between the two. Perineuriomas are produced by perineurial cells, while schwannomas, also referred to as neurilemmomas, are only formed by Schwann cells. A neurofibroma can arise from a variety of cell types, including endothelial cells, Schwann cells, fibroblasts, and perineural fibroblasts. A histopathological investigation is critical and remains the gold standard for detecting extraneural perineuriomas. Spindle cells grouped in whorls and fascicles are the hallmark of these tumors, with IHC staining typically positive for EMA and negative for S-100 protein [9,10]. In the absence of imaging, histopathology helps differentiate perineuriomas from other soft tissue tumors, such as schwannomas and neurofibromas, which are S-100 positive.

Excision with negative margins is the preferred treatment for extraneural perineuriomas. This approach is generally successful, with low recurrence rates and an excellent prognosis [11,12]. Extraneural perineuriomas have not been documented to undergo malignant transformation, which adds credence to their benign character.

## Conclusions

The histologic characteristics of many other benign and malignant soft tissue tumors overlap with those of perineurioma, a rare and underappreciated benign neoplasm. The right diagnosis is primarily based on the IHC profile and histologic findings, but in challenging cases, ultrastructural studies may be necessary. Surgical excision with negative margins remains the cornerstone of treatment, with excellent long-term outcomes and low rates of recurrence. Although cytogenetic and molecular genetic studies still have a limited role in the diagnosis of perineuriomas, they may be crucial in ruling out serious differential diagnoses like dermatofibrosarcoma protuberans and low-grade fibromyxoid sarcoma.
